# Missense variants in *CTNNB1* can be associated with vitreoretinopathy—Seven new cases of *CTNNB1*‐associated neurodevelopmental disorder including a previously unreported retinal phenotype

**DOI:** 10.1002/mgg3.1542

**Published:** 2020-12-22

**Authors:** Linda Z. Rossetti, Mir Reza Bekheirnia, Andrea M. Lewis, Heather C. Mefford, Katie Golden‐Grant, Kristina Tarczy‐Hornoch, Lauren C. Briere, David A. Sweetser, Melissa A. Walker, Elijah Kravets, David A. Stevenson, Georgette Bruenner, Jessica Sebastian, Julia Knapo, Jill A. Rosenfeld, Paul C. Marcogliese, Undiagnosed Diseases Network, Michael F. Wangler

**Affiliations:** ^1^ Department of Molecular and Human Genetics Baylor College of Medicine Houston TX USA; ^2^ Division of Genetic Medicine Department of Pediatrics University of Washington Seattle WA USA; ^3^ Department of Ophthalmology University of Washington Seattle WA USA; ^4^ Division of Medical Genetics and Metabolism Department of Pediatrics Massachusetts General Hospital Harvard Medical School Boston MA USA; ^5^ Department of Neurology Division of Neurogenetics Child Neurology Massachusetts General Hospital Boston MA USA; ^6^ Division of Medical Genetics Department of Pediatrics Stanford University Stanford CA USA; ^7^ Division of Medical Genetics Department of Pediatrics Cohen Children’s Medical Center Queens NY USA; ^8^ Division of Medical Genetics Department of Pediatrics UPMC Children’s Hospital of Pittsburgh Pittsburgh PA USA; ^9^ Jan and Dan Duncan Texas Children’s Neurological Research Institute Houston TX USA

**Keywords:** *CTNNB1*, developmental delay, familial exudative vitreoretinopathy, neurodevelopmental disorder with spastic diplegia and visual defects, visual defects

## Abstract

**Background:**

*CTNNB1* (MIM 116806) encodes beta‐catenin, an adherens junction protein that supports the integrity between layers of epithelial tissue and mediates intercellular signaling. Recently, various heterozygous germline variants in *CTNNB1* have been associated with human disease, including neurodevelopmental disorder with spastic diplegia and visual defects (MIM 615075) as well as isolated familial exudative vitreoretinopathy without developmental delays or other organ system involvement (MIM 617572). From over 40 previously reported patients with *CTNNB1*‐related neurodevelopmental disorder, many have had ocular anomalies including strabismus, hyperopia, and astigmatism. More recently, multiple reports indicate that these abnormalities are associated with the presence of vitreoretinopathy.

**Methods:**

We gathered a cohort of three patients with *CTNNB1*‐related neurodevelopmental disorder, recruited from both our own clinic and referred from outside providers. We then searched for a clinical database comprised of over 12,000 exome sequencing studies to identify and recruit four additional patients.

**Results:**

Here, we report seven new cases of *CTNNB1*‐related neurodevelopmental disorder, all harboring *de novo* variants, six of which were previously unreported. All patients but one presented with a spectrum of ocular abnormalities and one patient, who was found to carry a missense variant in *CTNNB1*, had notable vitreoretinopathy.

**Conclusions:**

Our findings suggest ophthalmologic screening should be performed in all patients with *CTNNB1* variants.

## Graphical abstract

Here, we report seven new cases of *CTNNB1*‐related neurodevelopmental disorder, all harboring de novo variants, six of which were previously unreported. All patients but one presented with a spectrum of ocular abnormalities and one patient, who was found to carry a missense variant in *CTNNB1*, had notable vitreoretinopathy. Our findings suggest that ophthalmologic screening should be performed in all patients with *CTNNB1* variants.

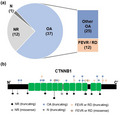

## 1 INTRODUCTION

*CTNNB1* (MIM 116806) encodes for beta‐catenin, an adherens junction protein responsible for maintaining the integrity between epithelial layers that also plays an important role in cell‐to‐cell signaling (Peifer, 1993). Somatic variants in *CTNNB1* have been associated with various solid tumors, including hepatocellular and colorectal carcinomas (Fearnhead et al., 2004; Taniguchi et al., 2002; Yoo et al., 2009). In 2012, de Ligt et al. (2012) described a total of three individuals with severe intellectual disability who were found on a large‐scale study of exome sequencing to have germline *de novo*, likely pathogenic variants in *CTNNB1*. These patients were also found to share other clinical features, including microcephaly, limited or absent speech, and lower extremity spasticity. Thirty‐three additional patients with similar phenotypes were then described in several subsequent case reports and series, including two unrelated individuals with distinct microdeletions of chromosome 3 containing the *CTNNB1* gene (Dubruc et al., 2014; Kharbanda et al., 2017; Kuechler et al., 2015; Tucci et al., 2014; Winczewska‐Wiktor et al., 2016). Many of the described patients also displayed a variety of ocular anomalies, including strabismus, hyperopia, and/or astigmatism, leading to the naming of the syndrome “neurodevelopmental disorder with spastic diplegia and visual defects” (NEDSDV, MIM 615075). However, no specific description of retinopathy in this condition was published until 2016, when a child with developmental delay who had a known disruptive variant in *CTNNB1* was also found to have familial exudative vitreoretinopathy (FEVR) and retinal detachment (Dixon et al., 2016). Since then, there have been additional patients described with FEVR, both with and without microcephaly and intellectual disability, who were found to have germline variants in *CTNNB1* (Coussa et al., 2020; Li et al., 2017; Panagiotou et al., 2017; Sun et al., 2019; Tipsuriyaporn et al., 2020; Wang et al., 2019), suggesting that vitreoretinopathy may truly be an important if not common feature in NEDSDV that could merit closer ophthalmologic surveillance in asymptomatic patients. Interestingly, all of the previously reported cases of NEDSDV involving vitreoretinopathy have been associated with truncating (nonsense, frameshift) or splicing variants in *CTNNB1*. Here, we report an additional seven patients with *de novo* previously unreported variants in *CTNNB1*, including two patients with vitreoretinopathy. One of these patients is the first, to our knowledge, to exhibit both the neurodevelopmental and ocular phenotype with a missense, single amino acid substitution variant. All variants were Sanger confirmed.

## 2 METHODS

We gathered a cohort of three patients with *CTNNB1*‐related neurodevelopmental disorder, recruited from both our own clinic and referred from outside providers. We then searched for a clinical database comprised of over 12,000 exome sequencing studies to identify and recruit four additional patients.

Patient whole‐exome sequencing was performed at multiple different laboratories. Patients 1 and 2 had sequencing performed by GeneDx with capture via GeneDx proprietary system and sequencing completed on an Illumina platform. XomeAnalyzer was used for data alignment and filtering, and coverage ranged from a mean depth of 85x‐110x, with 98.5% of reads having at least 10x coverage. Patients 3, 4, 6, and 7 had sequencing performed at Baylor Genetics using biotin‐labeled VCRome 2.1 in‐solution exome probes for capture. Sequencing was completed on an Illumina platform, and Mercury 1.0 was used for data alignment and filtering. The mean depth of coverage was >100x, with >95% of reads having at least 20x coverage. Patient 5 had sequencing performed at ARUP laboratories with capture using Agilent SureSelectXT liquid RNA based probes. Data analyses for all patients was completed per ACMG guidelines as previously described in Richards et al. (2015), using resources such as ExAC, EVS, SIFT, PolyPhen‐2, and 1000 Genomes Browser.

### 2.1 Analyses of *CTNNB1* were based on transcript NM_001904.4

#### 2.1.1 Ethical compliance

All patients were enrolled in an institutional review board‐approved research study after informed consent had been obtained. This study conforms to all institutional and international ethical standards.

## 3 CASE REPORT

### 3.1 Patient 1

Patient 1 is a 23‐month‐old male who was born at 36 weeks 5 days gestation. Pregnancy had been complicated by oligohydramnios. He was noted to be microcephalic at birth and had poor feeding/poor weight gain. He was globally developmentally delayed. An MRI of the brain was structurally normal; an MRI of the spine showed possible tethered cord, for which the patient underwent surgery. On exam, he was hypotonic in the trunk and upper extremities but spastic in the lower extremities. On ophthalmologic exam, he had intermittent exotropia with suspected amblyopia of the right eye. He also had mild hyperopia. Chromosomal microarray analysis (CMA) and mitochondrial DNA sequencing were normal. Trio whole‐exome sequencing detected a *de novo* nonsense variant in *CTNNB1* (c.1962T>A, p.Y654X) that was thought to be diagnostic.

### 3.2 Patient 2

Patient 2 is a 7‐year‐old male whose delivery was induced due to poor fetal growth. Pregnancy had been complicated by gestational diabetes. He was noted to be microcephalic at birth and continued to have poor weight gain and short stature in childhood. He was globally developmentally delayed. He exhibited repetitive behaviors and occasionally bit and pinched other people. He also had a diagnosis of restless leg syndrome. On exam, he was noted to have bradykinesia and toe walking, as well as hypertonia in the lower extremities. An MRI of the brain was structurally normal. On ophthalmologic exam, he had mild intermittent esotropia, mild hyperopia, and mild astigmatism. CMA and acylcarnitine profiles were normal. Trio whole‐exome sequencing detected a *de novo* frameshift variant in *CTNNB1* (c.776_777delTC, p.L259PfsX11) that was thought to be diagnostic.

### 3.3 Patient 3

Patient 3 is an 11‐year‐old female who was born at term after being noted to have poor growth in utero. She measured small for gestational age at birth and was microcephalic. No feeding issues were noted, but she did have constipation. She had gross and fine motor delays, and some articulation issues but otherwise normal speech development. She exhibited repetitive behaviors of hand‐wringing and nail‐picking. On exam, she was overall hypotonic but had spasticity in the lower extremities bilaterally. She also had a duplicated left thumb. From an ophthalmologic perspective, she had a history of retinal detachment repair and lensectomy in infancy (right eye) and early childhood (left eye), following retinal detachment thought to be secondary to FEVR, with mild microcornea, aphakic glaucoma, and legal blindness in the right eye, and total retinal detachment with no light perception in the left eye. CMA, chromosomal breakage studies and urine organic acids were normal. Trio whole‐exome sequencing detected a *de novo* missense variant in *CTNNB1* (c.1723G>A, p.G575R) that was thought to be diagnostic. The variant was predicted to be “probably damaging” by in silico analysis using PolyPhen‐2, as well as “disease causing” by MutationTaster. In addition, the variant was absent from the gnomAD population database (https://gnomad.broadinstitute.org/ accessed 8/31/2020).

### 3.4 Patient 4

Patient 4 is a 3‐year‐old female who was noted to be small for gestational age and microcephalic at birth after a reportedly uncomplicated prenatal course. She had global developmental delay, feeding aversion, and poor weight gain. In addition, she was diagnosed with type I diabetes. On exam, she was hypotonic though her tone did seem to improve as she grew older. An MRI of the brain was unremarkable, but on spectroscopy, she demonstrated an abnormal lactate peak. Her ophthalmologic exam was notable for bilateral tractional vitreoretinopathy with an history of bilateral retinal detachments as well as strabismus, hyperopia, and astigmatism. CMA was normal. Trio whole‐exome sequencing detected a deletion‐insertion variant in *CTNNB1* (c.1016_1025delinsT, p.T339_R342delinsI) that was thought to be diagnostic. Of note, this patient's retinal phenotype was previously reported in the literature (Tipsuriyaporn et al., 2020).

### 3.5 Patient 5

Patient 5 is a 4‐year‐old male who was born prematurely at 24 weeks of gestation. He was discharged from the neonatal intensive care unit at 84 days of life. Microcephaly was not noted at birth, but did develop as he progressed into early childhood. He had feeding difficulties, reflux, silent aspiration, and constipation. He also underwent tonsillectomy and adenoidectomy for sleep apnea. He had global developmental delay and was eventually diagnosed with autism spectrum disorder. He also exhibited some self‐injurious behaviors. On exam, he had truncal hypotonia with hypertonicity in the distal extremities. He also had some choreiform movements and intention tremor. An MRI of the brain was unremarkable. From an ophthalmologic perspective, he was known to have retinopathy of prematurity in addition to cortical visual impairment and strabismus. CMA and methylation studies for Angelman and Prader‐Willi syndromes were negative. Trio whole‐exome sequencing detected a *de novo* frameshift variant in *CTNNB1 *(c.1665dupG, p.T556fs) that was thought to be diagnostic.

### 3.6 Patient 6

Patient 6 is a 5‐year‐old male who was noted to be microcephalic at birth. He had a global developmental delay with absent speech. He also had a diagnosis of mild asthma. Physical exam was notable for hypotonia with spasticity in the lower extremities. CT of the head and EEG were normal. His ophthalmologic exam was normal. CMA detected loss of heterozygosity on chromosomes 1 and 11 but otherwise no copy number variants were detected. Methylation studies for Angelman and Prader‐Willi syndromes were negative. Rett syndrome testing was also negative. Trio whole‐exome sequencing detected a *de novo* frameshift variant in *CTNNB1 *(c.214_215del, p.Q72fs) that was thought to be diagnostic.

### 3.7 Patient 7

Patient 7 is an 8‐year‐old male who was noted to have intrauterine growth restriction prenatally (as part of a twin gestation). He was microcephalic at birth and was noted to have a murmur. Echocardiogram showed dysplastic and bicuspid pulmonary valve that is now status post valvuloplasty. He has a global developmental delay, absent speech, and self‐injurious behaviors, as well as a history of screaming spells. In addition, he had feeding issues and chronic diarrhea and was diagnosed with failure to thrive. On exam, he was hypotonic. Brain MRI showed only microcephaly and brachycephaly and EEG was normal. Ophthalmologic exam was notable for hyperopia, astigmatism, and esotropia of the left eye. CMA, chromosome analysis, and targeted testing for Noonan syndrome were normal. Trio whole‐exome sequencing detected a *de novo* frameshift variant in *CTNNB1 *(c.1005delA, p.K335fs) that was thought to be diagnostic.

## 4 DISCUSSION

**FIGURE 1:**
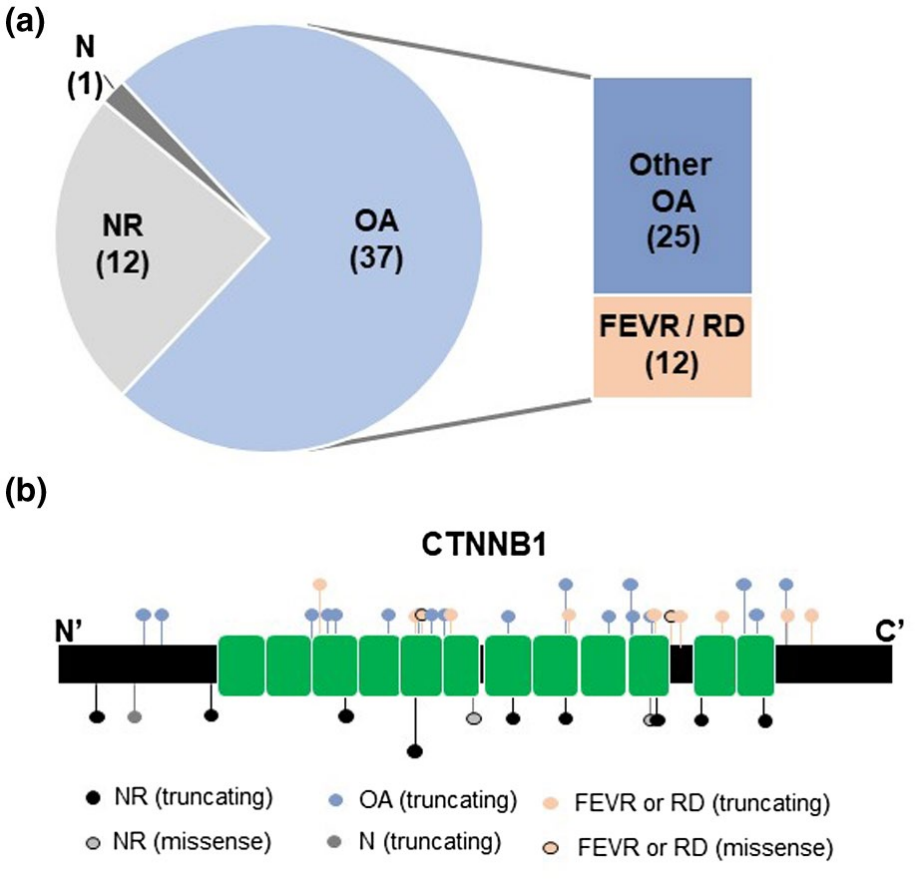
(a) Proportion of patients reported in literature with ocular phenotype. OA, ocular anomaly; FEVR, familial exudative vitreoretinopathy; RD, retinal detachment; NR, not reported; N, normal. (b) Schematic showing the distribution of reported variants in *CTNNB1*, type of variant, and associated ocular phenotype, if reported. Note there is no correlation between variant location, type of variant, and ocular phenotype. Longer line represents *n* = 2

Of the seven patients with *CTNNB1*‐related NEDSDV we report here, all had microcephaly and developmental delay, five had truncal hypotonia, and four had spastic diplegia, as would be expected given the cardinal features of the condition. In addition, all but one had some kind of ocular anomaly, with two patients having non‐prematurity‐related retinopathy with retinal detachment (Table 1). There are now multiple cases of *CTNNB1*‐related neurodevelopmental disorder or NEDSDV and some kind of vitreoretinopathy in the literature. Including patients from our cohort, there are now twelve individuals (of 50) with vitreoretinopathy (Table S1), and 37 total with some kind of ocular anomaly reported (Figure 1a). At present, there is no discernable genotype‐phenotype pattern as the retinopathy‐associated variants seem to be scattered throughout the gene, and include all variant types, including truncating, splice site, and missense, as we report here (Figure 1b). It is possible that there are other genetic loci, epigenetic modifiers, somatic events occurring the in same pathway, or environmental triggers that contribute to this particular complication, with pathogenic variants in *CTNNB1* creating a sensitized genetic background for its development. While vitreoretinopathy is not seen in the majority of these patients, it has now been reported in just under 25% of patients. Therefore, vitreoretinopathy has presented frequently enough that it seems prudent to recommend all individuals diagnosed with NEDSDV are routinely surveilled by an ophthalmologist with particular attention being paid to the possibility of retinopathy and signs of early retinal detachment in order to preserve as much vision as possible for these already complex patients .

**Table 1:** Summary of the variants and clinical characteristics of the patients reported here

Patient	1	2	3	4	5	6	7	Total
Variant	c.1962T>A, p.Y654X	c.776_777delTC, p.L259Pfs*11	c.1723G>A, p.G575R	c.1016_1025delinsT, p.T339_R342delinsI	c.1665dupG, p.Thr556fs	c.214_215del, pQ72fs	c.1005delA, p.K335fs	
Genomic coordinates (hg19)	3:41278086	3:41267190‐41267191	3:41277254	3:41268776‐41268785	3:41275771	3:41266216	3:41268763	
Inheritance	De novo	De novo	De novo	De novo	De novo	De novo	De novo	
Sex	M	M	F	F	M	M	M	
Age at diagnosis	20 months	6 yo	6 yo	2 yo	23 months	12 months	3 yo	
Microcephaly	+	+	+	+	+	+	+	7/7
Developmental delay	+	+	+	+	+	+	+	7/7
Spastic diplegia	+	+	+	NR	+	+	NR	5/5
Truncal hypotonia	+	NR	+	+	+	+	+	6/6
Hyperopia	+	+	+	+	−	−	+	5/7
Strabismus	+	+	+	+	+	−	+	6/7
Astigmatism	−	+	+	+	−	−	+	4/7
Retinal detachment	−	−	+	+	−	−	−	2/7
Other eye findings	None	None	FEVR	Bilateral tractional vitreoretinopathy	Retinopathy of prematurity	None	None	

Abbreviation: yo, years old; NR, not reported.

## CONFLICT OF INTEREST

The authors do not have any relevant conflicts of interest to report.

## AUTHORS’ CONTRIBUTION

Linda Z. Rossetti: original draft preparation (lead), review and editing (lead), investigation (equal). Mir Reza Bekheirnia: resources (equal), review and editing (supporting). Andrea M. Lewis: resources (equal), investigation (equal), review and editing (equal). Heather C. Mefford: resources (equal), review and editing (equal). Katie Golden‐Grant: resources (equal), investigation (equal), review and editing (equal). Kristina Tarczy‐Hornoch: investigation (equal), review and editing (supporting). Lauren C. Briere: resources (equal), investigation (equal), review and editing (equal). David A. Sweetser: resources (equal), review and editing (equal). Melissa A. Walker: resources (equal), review and editing (supporting). Elijah Kravets: resources (equal), investigation (equal). David A. Stevenson: resources (equal), review and editing (equal). Georgette Bruenner: resources (equal), investigation (equal). Jessica Sebastian: resources (equal), investigation (equal), review and editing (equal). Julia Knapo: resources (equal), investigation (equal). Jill A. Rosenfeld: resources (lead), review and editing (equal). Paul C. Marcogliese: visualization (lead), review and editing (equal). Michael F. Wangler: supervision (lead), investigation (equal), review and editing (equal).

## Supporting information

Supplementary Material

Table S1

Supplemental Document S1
