# Supplementary material for: Missense variants in CTNNB1 can be associated with vitreoretinopathy—Seven new cases of CTNNB1‐associated neurodevelopmental disorder including a previously unreported retinal phenotype
Source: Mol Genet Genomic Med. 2020 Dec 22;9(1):e1542. doi: 10.1002/mgg3.1542 (PMC7963417; doi:10.1002/mgg3.1542)
Supplement: Supplementary file 2 — Table S1 [file MGG3-9-e1542-s003.docx]

| **Patient ID** | **Variant** | **Sex** | **AGE AT DIAGNOSIS** | **PRENATAL ANOMALIES** | **GROWTH** | **DEVELOPMENT** | **BEHAVIOR** | **PERSONALITY** | **IQ** | **NEURO** | **EYE** | **FACIAL DYSMORPHISMS** | **FEEDING/GI ISSUES** | **CARDIAC ISSUES** | **RENAL/GU ISSUES** | **MSK** | **OTHER HEALTH ISSUES** | **OTHER GENETIC TESTING** |
| --- | --- | --- | --- | --- | --- | --- | --- | --- | --- | --- | --- | --- | --- | --- | --- | --- | --- | --- |
| Tucci 1 | p.Gln309* | M |  |  | Microcephaly (0.1th %ile), height 50th %ile, weight 3rd %ile | Gross motor delay (sat at 18mo, not walking at 30mo, walked at 4.5yo); no speech at 30mo but communicated with pictograms, had some sentences with poor articulation at 4.5yo |  | Happy | 72 at 4yo | MRI brain at 1yo with hypoplasia of corpus callosum | Hypermetropia with vision impairment | Full tip of nose, thin upper lip | Difficulties w/ chewing, swallowing |  |  | Hypotonia, broad big toes | None | SNP array (normal), PWS testing (normal), sequencing for CREBBP and EP300 (normal) |
| Tucci 2 | p.Ser425Thrfs*11 | F |  |  | Microcephaly (0.6th %ile), height 0.6th %ile, weight 50th %ile | Regression at 6mo with very slow progression following; sat at 2yo, crawled at 3yo, able to walk at 12yo with support; first words between 9-10yo | Aggression, automutilation, fecal smearing |  | Mod-severe ID at 29yo |  | NR | Full tip of nose, thin upper lip |  |  |  | Hypotonia at 6mo with progressive spasticity/hypertonia, fleshy hands with broad fingers, severe scoliosis | None | SNP array (normal), Rett-like testing including MECP2, FOXG1, CDKL5 (normal), sequencing for CREBBP and EP300 (normal), metabolic screen in blood/urine (normal) |
| Tucci 3 | p.Arg515* | F |  |  | Microcephaly (0.6th %ile), height <25th %ile, weight 50th %ile | Gross motor delay (walked only with walking frame) with progressive decline in motor skills leading to inability to walk at 50yo; no speech but able to use sign language; gradual decline in cognition |  | Cried a lot as infant |  |  | Slight atrophy of R optic nerve and moderate hypoplasia of L optic papilla | Full tip of nose, thin upper lip | Slow to feed as neonate, progressive swallowing difficulty |  |  | Progressive spasticity | None | SNP array from blood (normal), SNP array from buccal cells wth mosaic gain in 2q34, metabolic screen from blood (normal) |
| Tucci 4 | p.Gly236Argfs*35 | F |  |  | IUGR/SGA, microcephaly (<0.6th %ile), height 25-50th %ile, weight <0.6th %ile | Developmental delay since 6mo; sat without support at 12mo, walked at 4.5yo with support of walking frame; babbled at 3yo, speaking in simple sentences at 14yo and able to read simple words; had features of ASD; not entirely toilet trained |  | Excessive crying as infant | 65 at 7yo | Temperature dysregulation as infant | Strabismus s/p repair at 13mo | Full tip of nose, thin upper lip | Feeding difficulties as infant |  |  | Hypotonia, spastic diplegia of lower extremities, scoliosis in puberty | Recurrent URIs in infancy | SNP array (normal), Angelman methylation and UBE3A (normal), Rett syndrome MECP2 (normal), metabolic screen in blood/urine (nondiagnostic) |
| Tucci 5 | p.Thr551Met |  |  |  |  | ASD |  |  |  |  | NR |  |  |  |  |  |  |  |
| Dubruc 1 | 333 kb deletion on 3p22 | F | 5.5yo | IUGR | IUGR, BW -1.5 SD, length -2 SD, FOC -2 SD; postnatal microcephaly | Delayed; sat independently at 14mo, stood at 30mo, walked with aid at 3yo. Normal fine motor skills. 50 word vocabulary (with orofacial dyspraxia) and able to put 2 words together, receptive language > expressive | Hyperactivity | Friendly and sociable |  | Brisk polykinetic DTRs, +Babinski; brain MRI at 15mo with mild dilation of ventricles, normal spinal MRI and EEG | Significant hyperopia | Dysmorphic features; thin, sparse, fair hair; fair skin; thin lips; low-set ears | Abdominal US normal | ECHO normal |  | Progressive ataxia, spasticity, truncal hypotonia, sacral dimple, hyperextensible joints |  | Metabolic evalution (PAA, creatine metabolism, UOA) normal, chromosomes and fragile X normal, Angelman methylation normal |
| Kuechler 1 | p.Glu642Argfs*6 | M |  | Single umbilical artery | Normal weight/length at birth, primary microcephaly (-2.79 SD) | Started walking unsupported at age 8yo, toilet trained, speaks few single words with better comprehension, communicates with gestures | Good social interaction, outbursts of temper tantrums or crying, self-biting |  |  | Normal brain MRI, spine MRI with syringomyelia, broad based gait | Strabismus at 6mo, hyperopia | Hypotelorism, long/flat philtrum, thin upper lip vermilion |  |  |  | Hypertonia of the legs, broad hands with short distal phalanges, pes cavus, 2-4 toe syndactyly | Frequent respiratory infections |  |
| Kuechler 2 | p.Arg474* | F | 5yo |  | Normal weight/length at birth, primary microcephaly (-2.1 SD) | Severe delay; sat at 18mo, crawled at 23mo, pulled to stand at 33mo. | Stereotypic movements, no interest in toys | Generally friendly but occasional temper tantrums |  | No seizures, normal brain MRI | Hyperopia (+7/+6) | Broad nasal tip, flat occiput | Feeding difficulties, frequent vomiting, constipation | ECHO normal |  | Hypotonia of trunk and hypertonia of legs, tapered fingers |  |  |
| Kuechler 3 | c.1683+1G>A | F |  |  | Normal at birth, later developed microcephaly (-2.2 SD) | Motor delay, speech delay | Temper tantrums, crying, self-biting, little eye contact |  |  | Normal MRI brain/spine except for lumbosacral epidural lipoma | NR | Small nasal alae, broad nasal tip, long philtrum, small upper lip vermilion, deep-set ears, micrognathia |  |  |  | Hypotonia with peripheral hypertonia, hip dysplasia, pectus excavatum | Skin problems/eczema | Metabolic screening normal |
| Kuechler 4 | p.Leu251* | F |  |  | Normal at birth, later developed microcephaly (-2.1 SD) that improved with time | Motor delay, speech delay; walked at 10yo | Auto-aggression, self-injury, stereotypic movements |  |  | Toe-walking; normal MRI/EEG | Strabismus | Flat midface, small nasal alae, broad nasal tip, long/flat philtrum, high arched palate, small and low set ears |  |  |  | Hypotonia of trunk and hypertonia/dystonia of extremities, talipes | Sleep disturbance on Risperidone | Metabolic screening, neurotransmitters, CDG testing normal |
| Kuechler 5 | p.Tyr142Valfs*4 | F |  |  | Microcephaly (-2.94 SD) developed in first year of life | Motor delay, speech delay | Repetitive movements |  |  | Ataxic gait, MRI brain/spine normal, EEG normal | NR | Long face, small nasal alae, broad nasal tip, long and smooth philtrum, thin upper lip, diastema |  |  |  | Hypotonic | Sleep disturbances | Metabolic screening normal |
| Kuechler 6 (sib to K7) | p.Ser681* | F |  |  | Normal | Global developmental delay, ASD | Tics, aggression, oppositional behavior, occasional self-injury | Social | Mild ID, IQ 46-66 | Toe walking, tethered cord; MRI/CT brain normal | Strabismus, myopia | Upslanting palpebral fissures, boxy nasal tip, thin upper lip, long philtrum, narrow palate, small chin |  |  |  | Central hypotonia and peripheral hypertonia, scoliosis; prominent finger pads | ADHD, anxiety | Metabolic screening normal |
| Kuechler 7 (sib to K6) | p.Ser681* | M |  |  | Microcephaly (-3.2 SD), otherwise normal | Motor delay |  | Social | Borderline ID | Tethered cord, MRI brain normal | Bilateral esotropia | Dolichocephaly, upslanting palpebral fissures, boxy nasal tip, thin upper lip, long philtrum, small chin |  | PDA after birth |  | Central hypotonia and peripheral hypertonia | ADHD, hypo/oligodontia | Metabolic screening normal; also with variant in WNT10A a/w ectodermal dysplasia, likely accounting for hypo/oligodontia |
| Kuechler 8 | p.Cys419* | M |  | Microcephaly and ventriculomegaly seen on ultrasound | Progressive microcephaly (-4.8 SD), otherwise normal | Global developmental delay |  |  |  | Seizures at 2mo; MRI brain with enlarged lateral ventricles, dysgenesis of the corpus callosum, abnormal gyration of temporal lobe, absence of right fornix, and hypoplastic brainstem | Strabismus | Hypotelorism, upslanting palpebral fissures, epicanthus, long/flat philtrum, thin upper lip, prominent occiput | Feeding difficulties |  |  | Hypotonia of the trunk and hypertonia of the extremities |  | Karyotype from amniocentesis normal, metabolic screening normal |
| Kuechler 9 | p.Arg474* | M |  |  | Normal | Delayed, expressive language more delayed than receptive |  |  |  | Increased DTRs, possibly one febrile seizure, brain MRI with delayed myelination in frontal lobes | NR | Prominent metopic ridge, upslanting palpebral fissures, full nasal tip, long philtrum, thin upper lip, high palate, pointed chin | Feeding problems, difficulty swallowing/chewing |  |  | Hypertonicity in arms/legs since birth with axial hypotonia; deep palmar creases, mild clinodactyly of 5th fingers bilaterally, medial fatpat on feet | Difficulty falling asleep | Metabolic screening nonspecific |
| Kuechler 10 | p.Arg95* | F |  |  | Normal at birth, "low" FOC at 6mo | Delayed | Some self-injurious behavior, now improved |  |  | Abnormal toe-walking gait with ankles fixed in extension; normal EEG and normal MRI brain | R esotropia |  |  |  |  | "Stiff" per parents | Single umbilical artery noted at birth; cutis marmorata, hoarse voice | Normal biochemical studies |
| Kuechler 11 | p.Leu388Pro | M |  |  | Microcephalic (-3.3 SD) | Global developmental delay |  |  |  | Abnormal EEG with epileptiform activity, no clinical seizures | NR | Triangular face, deep-set eyes, small nares, prominent columella | Swallowing issues, texture aversion |  |  |  |  |  |
| Kuechler 12 | p.Glu642Valfs*5 | F |  |  | Normal | Delayed | Laughing spells at night, rages/tantrums; very short attention span; autism | Very friendly but poor eye contact |  | Generalized pyramidal symptoms; normal MRI brain x 2 | Strabismus, R sided amblyopia | Short philtrum, widely spaced teeth |  |  |  | Long slender fingers, long toes with sandal gap | Hoarse voice | Normal biochemical studies, normal peroxisomal investigations in cultured fibroblasts |
| Kuechler 13 | p.Gly34Asnfs*15 | M |  |  | Normal at birth, microcephalic by 5yo (-2.81 SD) | Motor delay | Limited concentration, sensitive to noises | Social and friendly |  | Exaggerated DTRs, +Babinski; MRI brain + MRS normal | NR |  |  |  |  | Axial hypertonia, talipes |  |  |
| Kuechler 14 | c.1081+1G>C | M |  | 2 vessel cord seen prenatally | Microcephalic since birth | Delayed motor development |  | Happy and social |  | Brisk DTRs, pronounced startle response to auditory/visual stimuli provoking breath holding spell | Photophobia | Asymmetric face (R bigger than L), sparse hair, deep-set eyes, upslanting palpebral fissures, broad nasal tip, long philtrum, thin upper lip vermilion, small teeth, small chin, large ears | Required NGT, then GT; on prokinetic therapy |  | Small penis, unilateral cryptorchidism | Low axial muscle tone and elevated peripheral muscle tone; leg asymmetry with R thigh circumference > L |  | Metabolic analyses normal, muscle biopsy without mitochondriopathy |
| Kuechler 15 | p.Ser425Thrfs*11 | F |  | IUGR | Low birth weight, microcephalic (-2.5 SD) | Motor delay, developmental regression with loss of words | Low tolerance for frustration | Happy and friendly |  | Dysmetria | Intermittent strabismus, poor coordination of eye movements | Thin hair, square face, deep-set eyes, stubby nose with broad nasal tip, thin upper lip vermilion |  |  |  | Truncal ataxia, hypertonic and spastic lower extremities |  | Metabolic screening normal |
| Kuechler 16 | 505 kb deletion at 3p22.1 | M |  | IUGR in 3rd trimester | Low birth weight and microcephaly at birth; short stature at 3yo with microcephaly (-4.09 SD) | Motor delay, speech delay |  | Generally happy demeanor |  | MRI brain with thinning of the corpus callosum, otherwise normal | R esotropia | R frontal hair upsweep, double posterior hair whorl, low set and posteriorly rotated ears, hypoplastic upper crus of inner helix, flat philtrum, thin upper vermilion |  |  |  | Persisently clenched hands at 3mo, increased tone in all extremities with dystonic posturing of upper extremities and scissoring of legs; thumbs adducted at rest; decreased tone in trunk |  | PAA, ACP, uric acid, AFP normal |
| Winczewska-Wiktor 1 | p.Q78* | M | 11yo |  | Mild microcephaly | Delayed speech, unable to walk unassisted at 20mo | Mild hyperactivity, verbal aggression, crying, sleep disturbance | Friendly, anxious | Mild ID | Ataxia; MRI with 12-13mm arachnoid cyst in posterior fossa and enlarged Sylvian fissure, otherwise normal; EEG showed mild slowing of background activity; exam with left hemiparesis with positive Babinski sign and mild dysarthria | Apraxia of upward gaze; astigmatism; hyperopia; normal fundus; periodic horizontal nystagmus; strabismus | Asymmetric face, right frontal hair upsweep, hypotelorism, deep set eyes, diastema, small teeth, short philtrum, thin upper lip vermilion, prominent columella, broad nasal tip, small alae nasi, hypoplastic upper crus of inner helix |  |  |  | Spastic paraplegia, truncal hypotonia; long slender fingers/toes, mild scoliosis | Episodic falls when startled by noise or touch (hyperekplexia); gynecomastia, inverted nipples | NPC1/NPC2 sequencing negative |
| Kharbanda 1 | p.Gln601* | M | 6yo | IUGR | Microcephalic | Globally delayed | Occasionally bites self/others; constantly active head rocking movements |  |  | No seizures | NR | Prominent nose, thin upper lip | Large appetite |  | S/p bilateral orchidopexies | Floppy at birth, truncal hypotonia | Single supernumerary upper incisor |  |
| Kharbanda 2 | p.Arg535* | M | 3yo | None | Microcephalic | Globally delayed |  |  |  | No seizures | Strabismus and hypermetropia | Thin upper lip, prominent lower lip, long smooth philtrum, small ears, brachycephaly |  |  |  | Peripheral spasticity and truncal hypotonia; dystonic posturing | Low oxygen sats at birth |  |
| Kharbanda 3 | p.Arg661* | F | 9yo | Microcephaly and IUGR | Microcephalic | Globally delayed | Some autistic traits and obsessional behavior |  |  | No seizures; poor balance, oromotor dyspraxia | NR | Widely spaced teeth, tented and thin upper lip, low set ears | Poor feeding and reflux |  |  | Sacral dimple, truncal hypotonia, slightly delayed bone age, hypermobile joints, 5th finger clinodactyly |  |  |
| Kharbanda 4 | p.Arg535* | M | 14yo | None | Normal | Globally delayed | Aggressive outbursts, self-harm |  |  | No seizures | Strabismus | Low set ears, short philtrum, thin upper lip, high arched palate, prominent chin | Truncal obesity |  | Absent left testis | Peripheral spasticity; brachydactyly, contractures at Achilles tendon | Persistent pulmonary hypertension at birth; poor sleep |  |
| Kharbanda 5 | p.Val349Alafs*9 | F | 11yo | IUGR | Progressive microcephaly | Speech and motor delay | Stereotypies, temper tantrums, aggressive toward family members |  |  | No seizures, ataxia | Strabismus, hypermetropia | Sparse hair over temples, periorbital fullness, slightly low set ears with fleshy lobes, full tip of nose |  |  |  | Truncal hypotonia | Had teeth at 3 months |  |
| Kharbanda 6 | p.Tyr333* | F | 27yo | None | Microcephalic | Globally delayed | Aggressive, temper tantrums, self-injurious behavior |  |  | No seizures, ataxia | NR | Unusual hair whorl pattern, thin lower lip, downslanting palpebral fissures, prominent columella, large chin, wide spaced teeth | Difficulty feeding |  |  | Floppy at birth, truncal hypotonia, peripheral spasticity, left clubfoot | Asthma |  |
| Kharbanda 7 | p.Arg474* | F | 13yo | None | Microcephalic | Globally delayed | Severe ADHD, aggressive, mouths objects and grinds teeth |  |  | No seizures | Bilateral strabismus | Deep-set eyes with darker periorbital skin, epicanthal folds, short prominent nose with bulbous tip, smooth philtrum, thin upper lip, high palate, micrognathia with pointed chin, hypertrophic gums |  |  |  | Peripheral spasticity and truncal hypotonia | Poor sleep |  |
| Kharbanda 8 | p.Gly268Trpfs*5 | F | 7yo | None | Microcephalic | Globally delayed, intermittent loss of skills | Autistic behaviors |  |  | No seizures, broad based gait | NR | Thin upper lip, slightly low columella |  |  |  | Peripheral spasticity and truncal hypotonia |  |  |
| Kharbanda 9 | p.Gln538* | F | 4yo |  | Microcephalic | Globally delayed | Autistic behaviors |  |  | No seizures, ataxia | Strabismus | Prominent eyes |  |  |  | Peripheral spasticity and truncal hypotonia | Congenital ichthyosiform erythroderma |  |
| Kharbanda 10 | p.Tyr333* | F | 9yo | None | Microcephalic | Globally delayed | Violent outbursts with difficulty expressing emotions |  |  | Possible absence seizures with normal EEG, small anterior fontanelle, irritable as infant | NR | Low set ears |  |  |  | Peripheral spasticity and truncal hypotonia; dystonic posturing |  |  |
| Li 1 | p.Gln558* | M | 15mo |  | Microcephaly, BW 3.6kg (normal) | Delayed; at 9mo could not roll or sit independently; no language at 15mo |  |  |  | MRI brain and EEG normal | Lack of light reaction, retinal detachement, lens and vitreous opacities bilaterally, falciform retinal folds, fundal hemorrhages | Low set ears, high arched palate | Abdominal US normal |  |  | Mild thumb adduction, hypertonia of extremities | Hearing normal | Metabolic workup, chromosome analysis normal |
| Sun 1 | c.999del | F | 3mo |  |  | Lack of head control at 15mo; no speech |  |  |  | CT at 5mo showing local osteolytic lesion on left parietal | Retinal detachment, iris posterior synechia, retrolenticular fibrotic mass |  |  |  |  | Scoliosis |  |  |
| Sun 2 | c.1104delT | F | 8mo |  | Microcephaly |  | Autism |  | Learning disabilities |  | Optic atrophy, macular dystrophy, temporal dragging of optic disc |  |  |  |  |  |  |  |
| Sun 3 | c.1738_1742delinsACA | M | 6mo |  |  |  |  |  |  |  | Corneal opacity, occlusion of pupil, temporal dragging of optic disc, retinal detachment |  |  |  |  |  |  |  |
| Sun 4 | c.1867C>T | M | 3mo |  |  |  | Autism |  | Learning disabilities |  | Corneal opacity, microcornea, occlusion of pupil, cataract, retrolenticular fibrotic mass, retinal detachment |  |  |  |  |  |  |  |
| Dixon | c.2112_2116dupAGAAC; p.P706QfsX31 | M | 22mo |  | Short stature | Delayed | Autistic behaviors |  |  | Lipomyelomeningocele | Bilateral hyperopic astigmatism, retinal detachment, exudative macular detachment; findings consistent with FEVR |  | FTT |  |  |  |  |  |
| Coussa | c.2046_2047del [p.Phe683Glnfs*9] | M | 2mo |  | Microcephalic | Mild motor delays |  |  |  |  | Bilateral retinal folds, findings consistent with FEVR |  |  |  |  |  |  |  |
| Tipsuriyaporn^ | (c.1016_1025delinsT,p.T339_342delinsI | F | 18mo |  |  |  |  |  |  |  |  |  |  |  |  |  |  |  |
| Panagiotou | c.1434_1435insC (p.Glu479Argfs*18) | M |  |  |  | Global developmental delay |  |  |  | Neuroimaging normal | Retinal detachment at 1mo; exam findings consistent with FEVR | Long face, prominent nasal tip |  |  |  | Skeletal survey normal |  |  |
| Wang 1 (daughter of 2) | c.734+1G>A | F | 27yo |  |  | Significant motor and speech delay |  |  | 28 on Wechsler test (severe ID) | Hyperreflexia, positive Babinski sign, ataxic gait; MRI brain normal, EEG mildly abnormal, EMG normal | Severe esotropia, L retinal detachment, eyeball atrophy |  |  |  |  | Involuntary rotation of the head, peripheral hypertonia, paroxysmal dystonia of head/neck |  | Ceruloplasmin normal |
| Wang 2 (mother of 1) | c.734+1G>A | F | 49yo |  |  |  |  |  | 40 on testing | Unremarkable MRI brain, EEG; ataxic gait | Esotropia, exudative vitreoretinopathy bilaterally |  |  |  |  |  |  |  |
| S1 | p.Tyr654* | M | 20mo | Oligohydramnios; elective c/s at 36w5d gestation | Microcephalic at birth, BW 5lbs 13oz | Global delay; rolling at 22mo but unable to sit independently and non-verbal | NR | NR | N/A | S/p tethered cord surgery at 21mo; normal MRI brain; MRI spine with mildly thickened fibrolipoma of the filum terminale, normal termination of conus, consider tethered cord | Mild hyperopia, strabismus; intermittent exotropia, suspected amblyopia of R eye | "Mildly dysmorphic" with full nasal tip | Poor feeding since birth, poor weight gain | NR | NR | Truncal and upper extremity hypotonia, lower extremity spasticity | NR | CMA and mitochondrial DNA sequencing normal |
| S2 | p.Leu259Profs*11 | M | 6yo | Gestational DM, induced due to poor growth | Microcephalic, poor weight gain, short stature | Global delay; walked at 2yo with assistance; at 5yo able to put sentences together but with poor speech coordination and knows shapes, colors, letters | Attention problems, restricted interests and repetitive behaviors, occasional biting/pinching of others | Sociable | NR | Slow movements and bradykinesia, difficulty sleeping, toe walking, normal MRI brain | Mild intermittent esotropia, mild astigmatism, mild hyperopia, strabismus | NR | Feeding issues at 6mo requiring NGT | NR | NR |  | Restless leg syndrome | CMA and ACP normal |
| S3 | p.Gly575Arg | F | 6yo | Unable to locate L kidney; poor growth in final month of gestation; born full term | BW= 2.5kg BL= 47cm Height, weight, and OFC consistently <3%ile since birth | Gross and fine motor delays; social immaturity; adaptive skill delay. Walked at 2y; Talked at 1.5y, then stopped speaking until 3y. Since then, speech has been on-track, with the exception of occasional stuttering. Speech therapy for stuttering determined this it be behavioral rather than developmental. Patient is bilingual- Russian and English | Repetitive behaviors- hand-wringing and nail-picking. At age 9, onset of stuttering when worried or emotionally worked up. | Shy, excited when talking or meeting a new person | NR | Imaging not performed | Retinal detachment in infancy (dx in Russia; ROP vs FEVR). Hyperopia, mild astigmatism, strabismus s/p correction. Current: no vision in L eye, glasses +10.50 sphere in both eyes with a +3.50 add in the right eye only- R eye vision 20/400 with glasses; s/p bilateral lensectomy;  Residual esotropia s/p strabismus surgery Light-sensitive | Deep-set eyes, upslanting palpebral fissures, nystagmus. Ears are notable for a simple helix and measure 5.5 cm bilaterally. Nose is somewhat thin and prominent. | Negative for feeding issues; +constipation | Murmur detected- normal echocardiogram | Left renal ectopia | Hypotonia Left thumb polydactyly- duplication of the distal phalanx with a nail Mild generalized osteoporosis and history of right distal fibula fracture Bilateral flexible flat feet with tight gastrocnemius and bilateral ankle valgus | NR | CMA (SNP) and Fanconi anemia chromosomal breakage study normal; normal urine oligosaccharides and urine organic acids |
| S4^ | p.Thr339_Arg342delinsIle | F | 2yo | None/unremarkable | SGA at birth, microcephaly, poor weight gain, short stature - weight & stature %iles increasing w/ age | Motor - sat unassisted (tripod) at 15 mo, army crawled at 13-14 mo, walked independently at 18mo, pincer grasp at 15mo Speech - markedly delayed, ~3 words at 27 mo, also a number of signs, receptive language much stronger | Feeding aversion | Unremarkable | N/A | Prior low tone, now low normal to normal. Brain MRIs largely unremarkable, MRS - definite abnormal lactate peak | Bilateral tractional vitreoretinopathy, h/o bilateral tractional retinal detachments, bilateral esotropia. Hyperopia, astigmatism, strabismus. | Not strikingly dysmorphic, but - small nose with slightly upturned tip, high arched palate with a bit of a midline ridge, full everted lower lip, ears slightly low set with small tragus and absent anti-tragus | Yes - feeding aversion & poor weight gain, improving with therapy | No | No | Initial low tone | Type 1 diabetes (+ family history) | CMA - normal, GeneDx dual mitochondrial genome panel - 1 het VUS in RANBP2, 1 het VUS, single het VUSs in VARS2, COQ6, & ATP7B; bone marrow failure sequencing & del/dup panel - single het VUS In SAMD9 |
| S5 | c.1665dupG; p.Thr556fs | M | 23mo | Born 24.5 weeks gestation; intermittent hemorrhaging early in pregnancy (6-8wks and then again right befor delivery). Normal prenatal ultrasounds; NIP screening normal. Neonatal period complicated by anemia of prematurity requiring two transfusions and respiratory distress (discharged from NICU at 84 days) | (BWt=0.65kg; BL 32cm; BOFC=21.5cm) (at 18mo: Wt=9.8kg; Ht=81.5cm; OFC=41.7cm) (at 23mo: Wt=10.8kg; Ht:84cm; OFC 43cm) (at 4y: Wt=16.3kg; Ht=98.4cm; OFC=45cm) | information from 23 month visit - global developmental delay: [rolled at 7 months; sat independently at approximately 20 months; not walking at 23months; no words at 23 months; babbles intermittently] Receives PT, OT, ST, vision services and feeding therapies; At 4 years is walking but mostly uses walker or stroller, has 200 words and knows numbers up to 30. | at 23 months some self-injurious behaviors (poke himself in eye to the point of bruising); diagnosed with autism spectrum disorder | Autism spectrum disorder; poor social interaction, insistence on sameness, fixated interests, decreased sensory input | NR | truncal hypotonia; distal hypertonicity; normal video EEG; diagnoses as having ataxic and spastic cerebral palsy; some choreiform movements of hands and feet with dysmetria/intention tremor. MRI of brain at 2 mo(non-specific findings of brain immaturity without actue intracranial abnormality | retinopathy of prematurity s/p laser surgery; cortical visual impairment; strabismus (s/p surgery); photophobia; Received patching; episodes of nystagmus after laser surgery but resolved | metopic prominence; large frontal upsweep of hair; curly hair; long eyelashes subjectively; slight lateral extension of the eyebrows; | History of silent aspiration with thin liquids; reflux; feeding difficulties; constipation | normal echocardiogram | none noted | wears AFOs | Required several sets of PE tubes; sleep apnea requiring tonsillectomy/adenoidectomy | normal SNP microarray; normal methylation for Angelman syndrome; on WES de novo variants of uncertain significance in the CPZ and TAOK2 genes |
| S6 | p.Q72fs | M | 1yo | Did not move a lot in utero, otherwise unremarkable | BW = 6lb 7oz, Microcephalic at birth (HC = 32 cm (5%)), Current head circ. = 46.5cm (-3.3 SD), height in 10th percentile, weight in 3rd percentile | Rolled at 1 year, sat at 2 years, pulled to stand at 3 years. Currently he can take steps with a walker and uses orthotics. He can understand simple commands, some two step commands. He has no words and only one word in sign language- "I want". He is not toilet trained. | No history of abnormal behaviors | MOC reports that he is always happy |  | severe intellectual disability, absent speech, spasticity of lower extremities, head CT w/out contrast- unremarkable, EEG- normal | Normal | microcephalic, thick helices in ears, high narrow arch in mouth | none reported | none reported | none reported | hypotonia, tight heel cords, spasticity of lower extremities, uses orthotics | mild asthma | Chromosome analysis normal, microarray analysis did not identify gains or losses but had areas of homozygosity between 1p31.3 to 1p31.1 and between 11q14.3 to 11q22.1, TORCH was normal, and methylation for prader-willi/Angelman syndrome were normal, Rett syndrome negative |
| S7 | p.K335fs | M | 3yo | assymetric IUGR (twin gestation) | BW = 5lb 3oz, weight as of 2018 = 35lbs, microcephalic at birth HC = 29cm, HC as of 2017 = 44cm(<3%), BL length = 46cm, length as of 2017 = 89cm | Head control - 6-7mos, Rolled over - 1yr, Crawled - 2yrs, pulled to stand - 26mos, global developmental delay, intelectual disability, non verbal | aggressiveness, self-abusive behaviors |  |  | severe developental delay, essential hypotinia, hx of screaming spells, with back arching, determined not to be seizures; normal EEG, brain MRI showing microcephaly and brachiocephaly | Optho note '13 - left eye esotropia, hyperopia, astigmatism | bitemporal narrowing, Single whorl noted. Eyebrows: prominent supraorbital ridge, bulbous nose | FTT, feeding issues improving with therapy, diarrhea with mushy and foul smelling stools | murmur noted after birth. echocardiogram showed a dysplastic bicuspid pulmonary valve, a bidirectional PDA and suprasystemic RV pressures. Pulmonary valve stenosis . s/p valvoplasty and cardiac catheterization | normal | able to walk which assistance, typically uses wheelchair. |  | Microarray - normal, karyotype - normal, noonan chip - normal |
