# Supplementary material for: Missense variants in CTNNB1 can be associated with vitreoretinopathy—Seven new cases of CTNNB1‐associated neurodevelopmental disorder including a previously unreported retinal phenotype
Source: Mol Genet Genomic Med. 2020 Dec 22;9(1):e1542. doi: 10.1002/mgg3.1542 (PMC7963417; doi:10.1002/mgg3.1542)
Supplement: Supplementary file 3 — Supplemental Document S1 [file MGG3-9-e1542-s001.docx]

**Members of the Undiagnosed Diseases Network**

Maria T. Acosta

Margaret Adam

David R. Adams

Pankaj B. Agrawal

Mercedes E. Alejandro

Justin Alvey

Laura Amendola

Ashley Andrews

Euan A. Ashley

Mahshid S. Azamian

Carlos A. Bacino

Guney Bademci

Eva Baker

Ashok Balasubramanyam

Dustin Baldridge

Jim Bale

Michael Bamshad

Deborah Barbouth

Pinar Bayrak-Toydemir

Anita Beck

Alan H. Beggs

Edward Behrens

Gill Bejerano

Jimmy Bennet

Beverly Berg-Rood

Jonathan A. Bernstein

Gerard T. Berry

Anna Bican

Stephanie Bivona

Elizabeth Blue

John Bohnsack

Carsten Bonnenmann

Devon Bonner

Lorenzo Botto

Brenna Boyd

Lauren C. Briere

Elly Brokamp

Gabrielle Brown

Elizabeth A. Burke

Lindsay C. Burrage

Manish J. Butte

Peter Byers

William E. Byrd

John Carey

Olveen Carrasquillo

Ta Chen Peter Chang

Sirisak Chanprasert

Hsiao-Tuan Chao

Gary D. Clark

Terra R. Coakley

Laurel A. Cobban

Joy D. Cogan

Matthew Coggins

F. Sessions Cole

Heather A. Colley

Cynthia M. Cooper

Heidi Cope

William J. Craigen

Andrew B. Crouse

Michael Cunningham

Precilla D'Souza

Hongzheng Dai

Surendra Dasari

Mariska Davids

Jyoti G. Dayal

Matthew Deardorff

Esteban C. Dell'Angelica

Shweta U. Dhar

Katrina Dipple

Daniel Doherty

Naghmeh Dorrani

Emilie D. Douine

David D. Draper

Laura Duncan

Dawn Earl

David J. Eckstein

Lisa T. Emrick

Christine M. Eng

Cecilia Esteves

Tyra Estwick

Marni Falk

Liliana Fernandez

Carlos Ferreira

Elizabeth L. Fieg

Laurie C. Findley

Paul G. Fisher

Brent L. Fogel

Irman Forghani

Laure Fresard

William A. Gahl

Ian Glass

Rena A. Godfrey

Katie Golden-Grant

Alica M. Goldman

David B. Goldstein

Alana Grajewski

Catherine A. Groden

Andrea L. Gropman

Irma Gutierrez

Sihoun Hahn

Rizwan Hamid

Neil A. Hanchard

Kelly Hassey

Nichole Hayes

Frances High

Anne Hing

Fuki M. Hisama

Ingrid A. Holm

Jason Hom

Martha Horike-Pyne

Alden Huang

Yong Huang

Rosario Isasi

Fariha Jamal

Gail P. Jarvik

Jeffrey Jarvik

Suman Jayadev

Jean M. Johnston

Lefkothea Karaviti

Emily G. Kelley

Jennifer Kennedy

Dana Kiley

Isaac S. Kohane

Jennefer N. Kohler

Deborah Krakow

Donna M. Krasnewich

Elijah Kravets

Susan Korrick

Mary Koziura

Joel B. Krier

Seema R. Lalani

Byron Lam

Christina Lam

Brendan C. Lanpher

Ian R. Lanza

C. Christopher Lau

Kimberly LeBlanc

Brendan H. Lee

Hane Lee

Roy Levitt

Richard A. Lewis

Sharyn A. Lincoln

Pengfei Liu

Xue Zhong Liu

Nicola Longo

Sandra K. Loo

Joseph Loscalzo

Richard L. Maas

Ellen F. Macnamara

Calum A. MacRae

Valerie V. Maduro

Marta M. Majcherska

Bryan Mak

May Christine V. Malicdan

Laura A. Mamounas

Teri A. Manolio

Rong Mao

Kenneth Maravilla

Thomas C. Markello

Ronit Marom

Gabor Marth

Beth A. Martin

Martin G. Martin

Julian A. Martínez-Agosto

Shruti Marwaha

Jacob McCauley

Allyn McConkie-Rosell

Colleen E. McCormack

Alexa T. McCray

Elisabeth McGee

Heather Mefford

J. Lawrence Merritt

Matthew Might

Ghayda Mirzaa

Eva Morava

Paolo M. Moretti

Marie Morimoto

John J. Mulvihill

David R. Murdock

Mariko Nakano-Okuno

Avi Nath

Stan F. Nelson

John H. Newman

Sarah K. Nicholas

Deborah Nickerson

Shirley Nieves-Rodriguez

Donna Novacic

Devin Oglesbee

James P. Orengo

Laura Pace

Stephen Pak

J. Carl Pallais

Christina GS. Palmer

Jeanette C. Papp

Neil H. Parker

John A. Phillips III

Jennifer E. Posey

Lorraine Potocki

Barbara N. Pusey

Aaron Quinlan

Wendy Raskind

Archana N. Raja

Deepak A. Rao

Genecee Renteria

Chloe M. Reuter

Lynette Rives

Amy K. Robertson

Lance H. Rodan

Jill A. Rosenfeld

Natalie Rosenwasser

Maura Ruzhnikov

Ralph Sacco

Jacinda B. Sampson

Susan L. Samson

Mario Saporta

C. Ron Scott

Judy Schaechter

Timothy Schedl

Kelly Schoch

Daryl A. Scott

Prashant Sharma

Vandana Shashi

Jimann Shin

Rebecca Signer

Catherine H. Sillari

Edwin K. Silverman

Janet S. Sinsheimer

Kathy Sisco

Edward C. Smith

Kevin S. Smith

Emily Solem

Lilianna Solnica-Krezel

Rebecca C. Spillmann

Joan M. Stoler

Nicholas Stong

Jennifer A. Sullivan

Kathleen Sullivan

Angela Sun

Shirley Sutton

David A. Sweetser

Virginia Sybert

Holly K. Tabor

Cecelia P. Tamburro

Queenie K.-G. Tan

Mustafa Tekin

Fred Telischi

Willa Thorson

Cynthia J. Tifft

Camilo Toro

Alyssa A. Tran

Brianna M. Tucker

Tiina K. Urv

Adeline Vanderver

Matt Velinder

Dave Viskochil

Tiphanie P. Vogel

Colleen E. Wahl

Stephanie Wallace

Nicole M. Walley

Chris A. Walsh

Melissa Walker

Jennifer Wambach

Jijun Wan

Lee-kai Wang

Michael F. Wangler

Patricia A. Ward

Daniel Wegner

Mark Wener

Tara Wenger

Katherine Wesseling Perry

Monte Westerfield

Matthew T. Wheeler

Jordan Whitlock

Lynne A. Wolfe

Jeremy D. Woods

Shinya Yamamoto

John Yang

Guoyun Yu

Diane B. Zastrow

Chunli Zhao

Stephan Zuchner
